# Magnolol enhances yak oocyte maturation and blastocyst quality by improving lipid metabolism and redox homeostasis through a PPARγ-dependent mechanism

**DOI:** 10.1186/s40104-026-01494-9

**Published:** 2026-10-02

**Authors:** Qiyong Zuo, Hui Zhang, Donglan Zhong, Tianhao Li, Yuejiao Liao, Yangyang Pan, Libin Wang, Sijiu Yu, Yan Cui

**Affiliations:** 1 College of Veterinary Medicine, Gansu Agricultural University, Lanzhou, 730070 China; 2 Gansu Innovation Centre for Livestock Embryo Engineering and Technology, Lanzhou, 730070 China

**Keywords:** Lipid metabolism, Magnolol, Oxidative stress, PPARγ, Yak oocytes

## Abstract

**Background:**

Reproductive efficiency in yaks is limited by the poor developmental competence of their oocytes during in vitro maturation (IVM). Magnolol (MAG), a natural polyphenolic compound with potent antioxidant activity, has been reported to regulate cellular metabolism and oxidative stress; however, its role in oocyte maturation and early embryonic development remains unclear. Therefore, this study investigated the effects and molecular mechanisms of MAG on yak oocyte maturation and developmental competence.

**Methods:**

Yak cumulus–oocyte complexes were cultured in IVM medium supplemented with different concentrations of MAG (0, 0.5, 1, 5, and 10 μmol/L). Oocyte maturation, cumulus expansion, oxidative stress indicators, mitochondrial function, apoptosis, and lipid metabolism were evaluated using fluorescence staining, qPCR, and immunofluorescence analysis. Network pharmacology and molecular docking were used to predict potential targets of MAG, and the peroxisome proliferator-activated receptor gamma (PPARγ) antagonist GW9662 was applied to verify the regulatory mechanism occurring during IVM and subsequent embryo development.

**Results:**

Based on the concentration-screening results, 1 μmol/L MAG was selected for subsequent mechanistic and embryo-development analyses. MAG supplementation significantly improved oocyte maturation and cumulus expansion, enhanced glutathione levels and mitochondrial membrane potential, and reduced reactive oxygen species accumulation and apoptosis. MAG also promoted lipid droplet accumulation and upregulated the expression of lipid metabolism–related genes, including *PPARG*, *CD36*, *FABP3*, and *FABP4*. Network pharmacology identified PPARγ as a key target of MAG. Pharmacological inhibition of PPARγ with GW9662 markedly reversed MAG-induced improvements in lipid metabolism, antioxidant capacity, and oocyte developmental competence. Furthermore, MAG increased blastocyst formation rate, total cell number, and CDX2 expression while reducing the blastocyst apoptosis.

**Conclusion:**

MAG improves yak oocyte quality and embryonic developmental competence by promoting lipid homeostasis, maintaining redox balance, and enhancing mitochondrial function. The beneficial effects of MAG are closely associated with the involvement of PPARγ signaling in the regulation of lipid homeostasis. These findings provide a potential strategy for improving the efficiency of yak assisted reproductive technologies.

**Graphical Abstract:**

**Figure:**
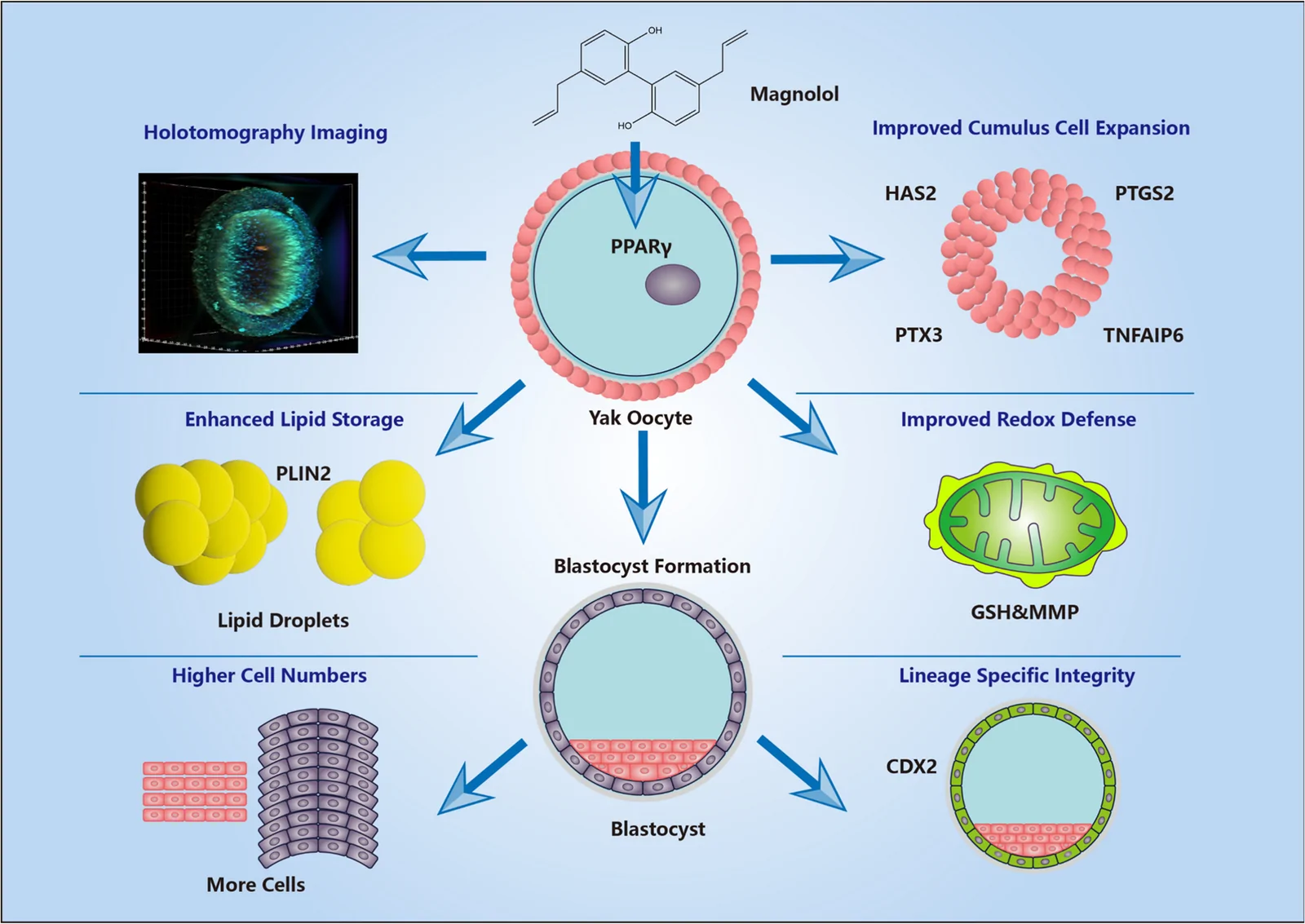

**Supplementary Information:**

The online version contains supplementary material available at https://doi.org/10.1186/s40104-026-01494-9.

## Introduction

The yak (*Bos grunniens*) is an ancient livestock species native to the Qinghai–Tibet Plateau and is highly adapted to long-term survival in high-altitude environments [1]. Yak-derived products, including meat, milk, hide, fiber, and dung, provide essential resources for daily life and constitute major sources of income for Tibetan and other pastoral communities [2–4]. Yaks are seasonally polyestrous animals, with estrus typically occurring between July and October [5]. However, their reproductive efficiency is remarkably low, commonly characterized by one calving every two years or two calvings within three years [6]. Consequently, yak populations have gradually declined in recent years, which not only restricts the improvement of local herders’ livelihoods but also poses a potential threat to livestock biodiversity in China. Therefore, the application of assisted reproductive technologies (ARTs) represents a promising strategy to improve yak reproductive performance and accelerate population expansion.

In vitro maturation (IVM) of oocytes is a fundamental component of ARTs; however, its efficiency in yak oocytes remains limited [7]. For example, compared with those from conventional cattle breeds, yak oocytes matured in vitro typically display lower developmental competence, characterized by decreased cleavage and blastocyst formation rates after in vitro fertilization (IVF) [8]. This limitation is mainly attributed to the lack of a physiological microenvironment [9]. Artificial culture systems are susceptible to multiple disturbances, including excessive accumulation of reactive oxygen species (ROS) and premature initiation of apoptosis. These adverse conditions trigger cellular stress responses and the generation of cytotoxic metabolites, ultimately compromising oocyte quality [10]. Poor oocyte quality is considered a primary cause of impaired fertilization and embryonic development, making it difficult to consistently obtain high-quality mature oocytes under in vitro conditions [11, 12]. Accumulating evidence suggests that supplementation of specific bioactive compounds into IVM systems can effectively improve oocyte maturation and subsequent embryonic development [13–15]. Various antioxidants, such as vitamins, selenium, inositol, carnitine derivatives, and polyphenolic compounds, have been reported to exert beneficial effects during oocyte maturation [16–18]. Magnolol (MAG), a principal active compound in magnolia officinalis, is a novel lignan compound typically found in the form of white or brown powder. Research has shown various beneficial effects of MAG, including anti-inflammatory [19], antioxidant [20], antilipid peroxidation [21], and cholesterol-lowering properties [22]. Previous studies have reported that MAG alleviates ovarian oxidative stress in aged laying hens by modulating the SIRT3/AMPK pathway [23]. MAG supplementation in pregnant mice improved systemic and placental antioxidant status by increasing superoxide dismutase (SOD) activity, reducing malondialdehyde (MDA) levels, and enhancing placental HO-1 expression [24]. Furthermore, MAG supplementation in laying hens alleviated ovarian oxidative stress and apoptosis and improved reproductive performance through regulating the gut–liver–ovary axis [25]. These findings indicate that MAG exerts beneficial effects in reproductive-associated tissues and supports its potential role in maintaining cellular homeostasis under oxidative stress conditions. However, the effects of MAG on oocyte maturation and early embryonic development have not yet been investigated.

The developmental competence of embryos largely depends on the intrinsic quality of oocytes, which is closely associated with cellular metabolic processes, particularly lipid metabolism [26]. Lipids are essential hydrophobic molecules involved in energy production, membrane biosynthesis, steroidogenesis, and signal transduction within cumulus–oocyte complexes (COCs). In oocytes and cumulus cells (CCs), lipids are primarily stored as triglycerides (TGs) within lipid droplets (LDs), serving as critical energy reserves for early embryonic development [27]. Previous studies have demonstrated that enhanced lipid metabolism improves oocyte quality and developmental potential in porcine models [28, 29]. Peroxisome proliferator-activated receptor gamma (PPARγ), a key member of the nuclear hormone receptor superfamily, plays a central role in regulating lipid metabolism, cellular differentiation, nutrient balance, and energy homeostasis [30–32]. Recent evidence indicates that activation of PPARγ promotes lipid metabolic activity, increases intracellular lipid content, and significantly improves oocyte maturation and blastocyst formation rates in sheep [33].

Therefore, in the present study, we aimed to investigate the effects of MAG on the IVM and early developmental competence of yak oocytes, with a particular focus on the potential involvement of PPARγ signaling in the beneficial effects of MAG on oocyte lipid metabolism. The aim of this study was to provide a theoretical basis for identifying effective compounds to optimize IVM systems and improve reproductive efficiency in yaks.

## Materials and methods

### Oocyte collection and IVM

Yak ovaries were transported to our laboratory within 2–3 h of being obtained from a local slaughterhouse. COCs were aspirated from follicles measuring 3–8 mm in diameter using an 18-gauge needle attached to a 10-mL syringe. COCs with homogeneous cytoplasm were selected under a stereomicroscope. Selected COCs were cultured in oocyte maturation medium (OMM) consisting of TCM-199 with Earle’s salts (Gibco, New York, USA) supplemented with glutamine (200 mmol/L), sodium pyruvate (100 mmol/L), follicle-stimulating hormone (0.2 U/mL), estradiol (2 μg/mL), gentamicin (0.01 g/mL), fetal bovine serum (10%, v/v), and epidermal growth factor (10 ng/mL). Cultures were maintained at 38.5 °C under 5% CO_2_ in humidified air for 24 h. According to the experimental design, MAG (0, 0.5, 1, 5, and 10 μmol/L; MedChemExpress, China) and the PPARγ antagonist GW9662 (1 μmol/L; MedChemExpress, China) were added to the maturation medium. After maturation, COCs were treated with 0.1% hyaluronidase to remove CCs. Oocyte maturation was assessed according to the extrusion of the first polar body, indicating progression to metaphase II (MII). The maturation rate was calculated as the percentage of oocytes reaching the MII stage. Unless otherwise indicated, all experiments were performed using three independent biological replicates. Each biological replicate consisted of oocytes collected from an independent ovary collection session, followed by independent IVM, IVF, and embryo culture procedures. Approximately 200 COCs meeting the selection criteria were included in each treatment group for each biological replicate. The number of oocytes, COCs, or embryos analyzed for each assay is specified in the corresponding Materials and Methods section. The independent biological replicate was considered the experimental unit for statistical analysis.

### IVF and embryo culture

After 24 h of IVM, oocytes were collected and partially denuded through treatment with 0.1% hyaluronidase (H3506, Sigma-Aldrich, USA) to remove excess CCs, leaving two layers of CCs intact. The oocytes were then transferred to BO-IVF medium (71004, IVF Bioscience, UK) and maintained at 38.5 °C under 5% CO_2_ in humidified air. Frozen–thawed semen was processed using Percoll density gradient centrifugation using PureSperm™ 40 and PureSperm™ 80 (Nidacon, Sweden). After centrifugation at 500 × *g* for 5 min, motile spermatozoa were recovered, washed, and resuspended to a final concentration of 2–4 × 10^6^ sperm/mL. Spermatozoa were added to BO-IVF droplets containing oocytes and co-incubated for 12–16 h at 38.5 °C under 5% CO_2_ and saturated humidity. Following fertilization, zygotes were cultured in BO-IVC medium (71005, IVF Bioscience, UK) at a density of 25 embryos per 100 μL droplets. Embryos were cultured at 38.5 °C under 6% O_2_, 5% CO_2_, and saturated humidity. For each biological replicate, approximately 100 zygotes per treatment group were cultured for embryo development. Cleavage was assessed at 24 h post-fertilization, and blastocyst formation was evaluated on d 7.5. Cleavage rate and blastocyst formation rate were calculated separately for each biological replicate. Cleavage rate was defined as the percentage of cleaved embryos relative to the total number of zygotes, whereas blastocyst formation rate was defined as the percentage of blastocysts relative to the total number of zygotes. All embryos were included in the calculation of developmental rates. For subsequent immunofluorescence and TUNEL analyses, only expanded d 7.5 blastocysts were selected to minimize variability associated with developmental stage and expansion status. Selected blastocysts were fixed in 4% paraformaldehyde for further analysis.

### Cumulus expansion index

Twenty-four hours after maturation, the cumulus expansion index (CEI) of COCs was scored on a scale of 0–4 according to the degree of cumulus cell expansion: 0, no expansion of CCs; 1, no expansion but CCs appeared spherical; 2, expansion limited to the outermost cumulus cell layers; 3, expansion of all cumulus cell layers except the corona radiata; and 4, complete expansion of all cumulus cell layers [34]. At least 30 matured COCs were assessed per replicate, and three independent biological replicates were performed for each treatment group.

### Immunofluorescence staining and quantification

Fixed samples were washed with PBST, permeabilized with 0.5% Triton X-100 (IT9100, Solarbio, China) for 25 min, and blocked with 10% bovine serum albumin (BSA) for 1 h at room temperature. Samples were then incubated overnight at 4 °C with primary antibodies against PPARγ (1:400; #AF6284, Affinity Biosciences), PLIN2 (1:200; #TC54764, Affinity Biosciences), HAS2 (1:200; #DF13702, Affinity Biosciences), PTGS2 (1:200; #AF7003, Affinity Biosciences), PTX3 (1:200; #13797-1-AP, Proteintech), TNFAIP6 (1:200; #AF5492, Affinity Biosciences), GPX1 (1:200; #ab22604, Abcam), SOD2 (1:200; #ab13533, Abcam), BCL2 (1:200; #bs-4563R, Bioss), BAX (1:200; #bs-0127 M, Bioss), CASP3 (1:200; #AF6311, Affinity Biosciences), GDF9 (1:200; #AF7535, Affinity Biosciences), BMP15 (1:400; #18982-1-AP, Proteintech), and CDX2 (ready-to-use; #AM392-5 M, BioGenex). After washing, samples were incubated with Alexa Fluor™ 594-conjugated donkey anti-rabbit IgG (H + L) or Alexa Fluor™ 488-conjugated donkey anti-mouse IgG (H + L) (1:800; Jackson ImmunoResearch) for 1.5 h at room temperature. Nuclei were counterstained with 4′,6-Diamidino-2-phenylindole (DAPI, SL7101, Coolaber, China). Rabbit IgG Isotype Control (1:100; #98136-1-RR, Proteintech) and Mouse IgG Isotype Control (1:100; #HY-P80757, MCE) were used as negative controls in place of the primary antibody, and no specific fluorescence signal was detected (Supplementary Material 1). Images were acquired using a DeltaVision™ Ultra imaging system (GE Healthcare Bio-Sciences Corp., USA) equipped with a 20 × objective. Z-stack images were collected throughout the entire oocyte, COCs, or blastocyst at 2.5 μm intervals. All images were analyzed using ImageJ software (NIH, USA) with identical acquisition settings, background correction procedures, thresholding criteria, and image-processing parameters within each experiment. For oocyte analyses, the region of interest (ROI) was defined as the entire oocyte area, including the nuclear region. Mean fluorescence intensity was quantified from the complete Z-stack projection. For HAS2, PTGS2, PTX3, and TNFAIP6 analyses, fluorescence quantification was restricted to the cumulus-cell compartment. Nine ROIs were evaluated per COC throughout the Z-stack images, and the average fluorescence intensity per pixel was calculated. For blastocyst analyses, expanded blastocysts from d 7.5 were included. Complete Z-stack images covering the entire embryo were acquired and analyzed. Total cell number was determined by counting all DAPI-stained nuclei, whereas trophectoderm (TE) cells were identified as CDX2-positive cells. Inner cell mass (ICM) cell number was calculated as total cell number minus TE cell number, and the ICM/TE ratio was subsequently determined for each blastocyst. In total, 15 oocytes, 15 COCs, or 10 blastocysts were evaluated per treatment group in each of three independent biological replicates.

### BODIPY 493/503 staining

BODIPY 493/503 staining was performed to assess intracellular lipid content in oocytes. Samples from each treatment group were fixed in 4% paraformaldehyde for at least 30 min at room temperature and subsequently incubated with BODIPY 493/503 working solution (10 μmol/L, 121207-31-6, MedChemExpress) for 25 min. Fluorescence images were acquired using a DeltaVision™ Ultra imaging system (GE Healthcare Bio-Sciences Corp., USA). Complete Z-stack images covering the entire oocyte were collected under identical imaging settings, including objective magnification, excitation intensity, exposure time, and detector parameters. Images were processed and analyzed using ImageJ software. ROI was defined as the entire oocyte area. Background fluorescence was measured from cell-free regions and subtracted from the corresponding fluorescence signals. Identical thresholding and image-processing parameters were applied to all samples. Intracellular lipid accumulation was quantified as the mean BODIPY fluorescence intensity within the entire oocyte ROI. A total of 15 oocytes were analyzed per biological replicate, with three independent biological replicates performed for each treatment group.

### TUNEL staining

DNA fragmentation was assessed using a TUNEL assay (Roche, Germany) according to the manufacturer’s instructions. Briefly, samples were washed in PBS, fixed in 4% paraformaldehyde (Wako, Japan), and permeabilized with 0.5% Triton X-100 (Sigma, Germany) for 40 min. Samples were then incubated with TUNEL reaction mixture at 37 °C for 1 h, followed by nuclear counterstaining with DAPI (Sigma, Germany) for 5 min. Fluorescence images were acquired using a DeltaVision™ Ultra imaging system (GE Healthcare Bio-Sciences Corp., USA). Negative controls were processed without terminal deoxynucleotidyl transferase, whereas positive controls were pretreated with 50 μg/mL DNase I (Sigma, Germany) for 30 min before TUNEL staining. For blastocyst analysis, only expanded blastocysts from d 7.5 were included. Complete Z-stack images covering the entire embryo were acquired and analyzed. Total nuclei were identified by DAPI staining, and apoptotic nuclei were identified as TUNEL-positive nuclei throughout the complete Z-stack. The numbers of total and TUNEL-positive nuclei were counted manually using FIJI/ImageJ software (NIH, USA). The apoptotic index was calculated as the percentage of TUNEL-positive nuclei relative to the total number of nuclei. A total of 10 blastocysts were analyzed per replicate, and three independent biological replicates were performed for each treatment group.

### Live-cell imaging

MII oocytes were separately stained with Monochlorobimane (10 μmol/L, 76421-73-3, MedChemExpress), JC-1 (10 μg/mL, 3520-43-2, MedChemExpress) and the ROS Assay Kit (50 μg/mL, S0033S, Beyotime) at 38.5 °C for 30 min to evaluate intracellular glutathione (GSH) levels, mitochondrial membrane potential, and intracellular ROS levels, respectively. After washing, fluorescence signals for GSH, mitochondrial membrane potential, and intracellular ROS levels were captured using an Olympus IX81 fluorescence microscope (Tokyo, Japan). Fluorescence intensity was quantified using ImageJ software (NIH, Bethesda, MD, USA). A total of 15 oocytes were analyzed per replicate, with three independent biological replicates.

### Determination of early apoptosis

**Fig. 1:**
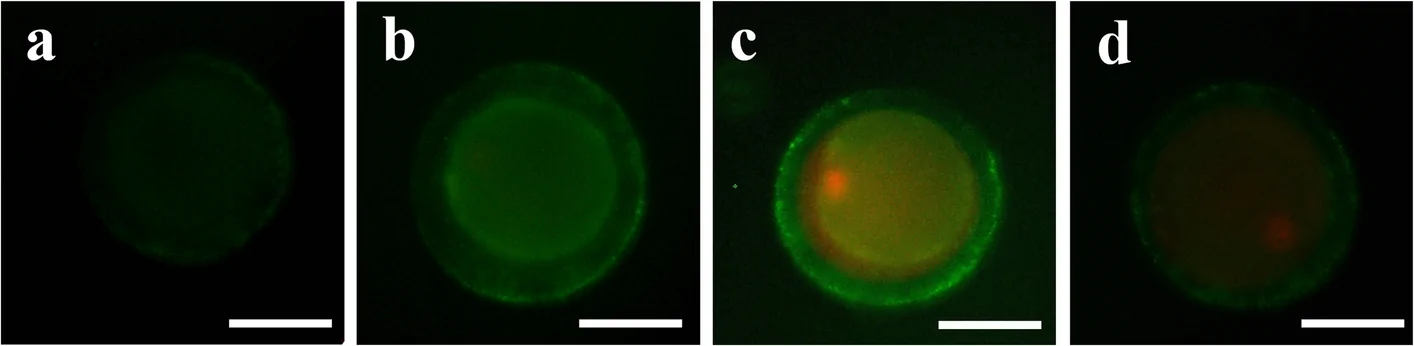
Representative images of early apoptosis detection in yak oocytes. **a** Viable oocytes, negative for Annexin V and PI signal. **b** Early apoptotic oocytes, positive for Annexin V green staining of the ooplasmic membrane and PI negative. **c** Non-viable oocytes, with Annexin V and PI positive signals. **d** Negative for Annexin V and positive for PI signal. Scale bar = 50 μm

Early apoptosis staining was performed using an Annexin V FITC-PI Apoptosis Detection Kit (HY-K1073, MCE) according to the manufacturer's instructions. Briefly, oocytes were stained for 15 min with Annexin-V/FITC and 100 µg/mL propidium iodide (PI) at 37 °C in the dark. After incubation, oocytes were washed thrice in PBS-PVA and mounted on slides. Samples were evaluated using an Olympus IX81 fluorescence microscope (Tokyo, Japan). Oocytes were classified into four groups (Fig. 1): (a) viable oocytes, negative for Annexin V and PI signal; (b) early apoptotic oocytes, positive for Annexin V green staining of the ooplasmic membrane and PI negative; (c) non-viable oocytes, with Annexin V and PI positive signals; and (d) negative for Annexin V and positive for PI signal. The early apoptosis rate was calculated as the percentage of Annexin V-positive and PI-negative oocytes relative to the total number of evaluated oocytes. At least 15 oocytes were assessed per replicate, and three independent biological replicates were performed for each treatment group.

### Quantitative real-time PCR

To ensure the accuracy of RNA-seq analysis and the validation of related genes, selected genes were confirmed by qPCR. Primers for each gene, synthesized by Shanghai Sangon Biotech Co., Ltd., were designed using the NCBI/Primer-Blast software, and sequence alignment was performed using BLASTN. The primer sequences, annealing temperatures, GenBank accession numbers, and amplification efficiencies are shown in Table 1. A non-RT control was included to assess genomic DNA contamination, and no amplification was observed in these controls. Primer specificity was initially validated by agarose gel electrophoresis, confirming a single amplicon of the expected size. In addition, melting curve analysis was performed at the end of each qRT-PCR run, and all reactions displayed a single peak, indicating specific amplification. Total RNA was extracted from yak oocytes, COCs, and blastocysts using the Arcturu™ PicoPure™ RNA Isolation Kit (Thermo Fisher, CAS #KIT12204). cDNA was synthesized using the GoScript™ Reverse Transcription Kit (Promega, Madison, USA), and samples were stored at −20 °C for later use. This mixture was then subjected to qPCR using the Roche 480 instrument (Roche, Basel, Switzerland) under the following conditions: pre-denaturation at 95 °C for 30 s, denaturation at 95 °C for 5 s, annealing at 60 °C for 34 s, and extension at 72 °C for 30 s, for a total of 45 cycles. To ensure reliable normalization under the present experimental conditions, the expression stability of beta-actin (*ACTB*) and TATA-box binding protein (*TBP*) was evaluated in MII oocytes, COCs, and blastocysts from all treatment groups (Control, MAG, MAG + GW9662, and GW9662) using NormFinder and BestKeeper. Based on the combined results of these two algorithms, *ACTB* and *TBP* were identified as stable reference genes and thus were selected as the benchmarks for data normalization. Relative mRNA expression levels were calculated using 2^−ΔΔCt^ method. The geometric mean of the Ct values of ACTB and TBP was used as the normalization factor, and the control group served as the calibrator for relative quantification. For qPCR analysis, samples were collected separately according to the experimental design. Each biological replicate consisted of 30 oocytes, 30 COCs, and 15 blastocysts, and three independent biological replicates were analyzed for each treatment group.

**Table 1:** Primer sequences and conditions for qPCR

Gene	Primer sequence (5'→3')	Temperature, °C	GenBank accession No.	Amplification efficiency, %
*ACTB*	F: CCGTGACATCAAGGAGAAG	56	XM_070362169.1	100
	R: AGGAAGGAAGGCTGGAAG			
*TBP*	F: GTCCAATGATGCCTTACGG	56	XM_005908677.2	98
	R: TGCTGCTCCTCCAGAATAGA			
*HAS2*	F: ACTCATTCCCGTATCCGTTTG	56	NM_174079.3	100
	R: TTCTTCCGCCTGCCACATT			
*PTGS2*	F: TTCTCGTGAAGCCCTATGA	58	NM_174445.2	99
	R: GAGGCAGTGTTGATGATTTT			
*PTX3*	F: GCTATCGGTCCATAATGCTTGT	57	NM_001076259.2	99
	R: CTTTCTTTGAATCCCAGGTGC			
*TNFAIP6*	F: CAAAGGAGTGTGGTGGTGTGTT	56	NM_001007813.2	102
	R: TTCAACATAGTCAGCCAAGCAA			
*BCL2*	F: TCGCCGAGATGTCCAGTCAAC	63	XM_070361474.1	101
	R: CACCCCGTCCCTGAAGAGC			
*BAX*	F: CGGCTGCTGGGCTGGATC	64	XM_005895371.2	100
	R: GGTGAGCGAGGCGGTGAG			
*CASP3*	F: AGACAGTGGTGCTGAGGATGAC	59	XM_014480600.2	99
	R: CCAGGTGCTGTAGAATATGCGTAC			
*PPARG*	F: GTCAGTTTCGCTCCGTGGAAG	59	XM_070383222.1	100
	R: TGGTCGTTCAAGTCAAGGTTCAC			
*CD36*	F: ATGTGCAGAATCCAGATGAAGTGAC	64	NM_001278621.1	99
	R: CGAACTCTGTATGTGTAAGGACCTC			
*FABP4*	F: AGAAGTGGGCGTGGGCTTTG	65	XM_005897260.3	100
	R: AGGTGCTTTCGGATTTAATGGTGAC			
*FABP3*	F: AGTTCGATGAGACCACAGCAGATG	63	XM_005906169.3	102
	R: CGTCAACCATTTCCCGCACAAG			
*BMP15*	F: CAGAGCCACTGTGGTTTACC	60	XM_005898488.2	99
	R: AGGAGGAGGAGAAAGGTCAG			
*GDF9*	F: CCAACAGAAGCCACCTCTACAAC	58	XM_005888179.3	98
	R: TTCCACAACAGTAACACGATCCAG			
*PLIN2*	F: CAGTGGTCAGTGGGCAGCAT	60	XM_005902268.3	100
	R: TGAGCACAGTGGGATTCATC			
*GPX1*	F: CAGTTTGGGCATCAGGAAAA	60	XM_005909375.3	99
	R: GGCACAGTCTGGGCCCACCA			
*SOD2*	F: GCTCTGCAGCCTGCGTTGAA	60	XM_070376974.1	100
	R: TGCACATTCAGGATAATCGGGC			

### Holotomographic imaging

Holotomographic imaging was performed using a Tomocube HT-X1 system (Tomocube, Republic of Korea), a quantitative phase-imaging microscope based on holographic tomography. The system employs LED illumination with a central wavelength of 450 nm to generate label-free three-dimensional images based on refractive index differences within yak oocytes. Due to their high refractive index, intracellular lipid structures were readily identified and quantified using this approach. Images were reconstructed and analyzed using TomoStudio™ software (v2.0, Tomocube). The lipid volume ratio, calculated as the ratio of total lipid droplet volume to total cell volume, was used to quantify intracellular lipid accumulation [35].

### Network pharmacology analysis

#### Identification of MAG targets

Potential molecular targets of MAG were retrieved from the Traditional Chinese Medicine Systems Pharmacology (TCMSP) database (https://www.tcmsp-e.com). MAG’s three-dimensional structure was obtained from the PubChem database (PubChem CID: 72300) and the target protein was predicted and sequenced based on the pharmacophore structure using the PharmMapper database (https://www.lilab-ecust.cn/pharmmapper). Using *Bos grunniens* as the species, the potential targets collected from the TCMSP and the PharmMapper databases were imported into the UniProt database (https://www.uniprot.org) to retrieve the validated drug target genes.

#### Identification of lipid metabolism disorder-related targets

To identify lipid metabolism disorder-related targets, the keyword “Lipid metabolism disorder” was used to retrieve genes from GeneCards, DrugBank, OMIM, DisGeNET, and the Therapeutic Target Database (TTD). After removing duplicate entries, the collected targets were integrated to generate a comprehensive lipid metabolism-related target dataset. These targets were subsequently intersected with the predicted MAG targets to identify potential regulatory targets associated with lipid metabolism.

#### Network construction and enrichment analysis

Overlapping targets between MAG-associated targets and lipid metabolism–related disease genes were identified through Venn diagram analysis. Protein–protein interaction (PPI) networks were constructed using the STRING database with the species restricted to *Bos taurus* and a confidence score ≥ 0.7. Network topology was analyzed and visualized using Cytoscape (v3.9.1) with the cytoNCA plugin to identify core targets; those with a degree ≥ 25 were selected. Gene Ontology (GO) functional annotation and Kyoto Encyclopedia of Genes and Genomes (KEGG) pathway enrichment analyses were conducted using the DAVID database. A comprehensive drug–target–pathway–disease network was constructed using Cytoscape.

### Molecular docking

The two-dimensional structure of MAG was downloaded from the PubChem database and optimized into a three-dimensional structure using Chem3D 19.0. The crystal structure of PPARγ (PDB ID: 6K0T), signal transducer and activator of transcription 3 (STAT3, PDB ID:5AX3), protein kinase cAMP-activated catalytic subunit alpha (PRKACA, PDB ID:6SNN) and estrogen receptor 1 (ESR1, PDB ID:6VPF) were obtained from the RCSB Protein Data Bank. Molecular docking was performed using AutoDock Vina following protein and ligand preparation with AutoDock Tools. Semi-flexible docking was applied, and binding affinity scores were calculated to assess the interaction between MAG and the core targets, including PPARγ, STAT3, PRKACA, and ESR1. The structure with the lowest binding free energy was selected for visualization by PyMOL.

### Statistical analysis

All experiments were performed with at least three biological replicates, and all values are presented as mean ± SEM. For each treatment group, the mean value obtained from all oocytes or embryos analyzed within one biological replicate was considered as one experimental unit for statistical analysis. Data were analyzed and plotted using GraphPad Prism 8, and Student's *t*-test was used for comparison between two independent samples (^*^P < 0.05, ^**^P < 0.01, and ^***^P < 0.001 for significant differences). The comparison of means among the multiple groups of data was analyzed by one‐way ANOVA. Tukey's multiple comparisons and different superscripts indicate significant differences (*P* < 0.05). For maturation, cleavage, and blastocyst development, percentages were first calculated for each independent biological replicate and the replicate values were used for statistical analysis.

## Results

### Effects of MAG on IVM and maturation-related gene expression in yak oocytes

**Fig. 2:**
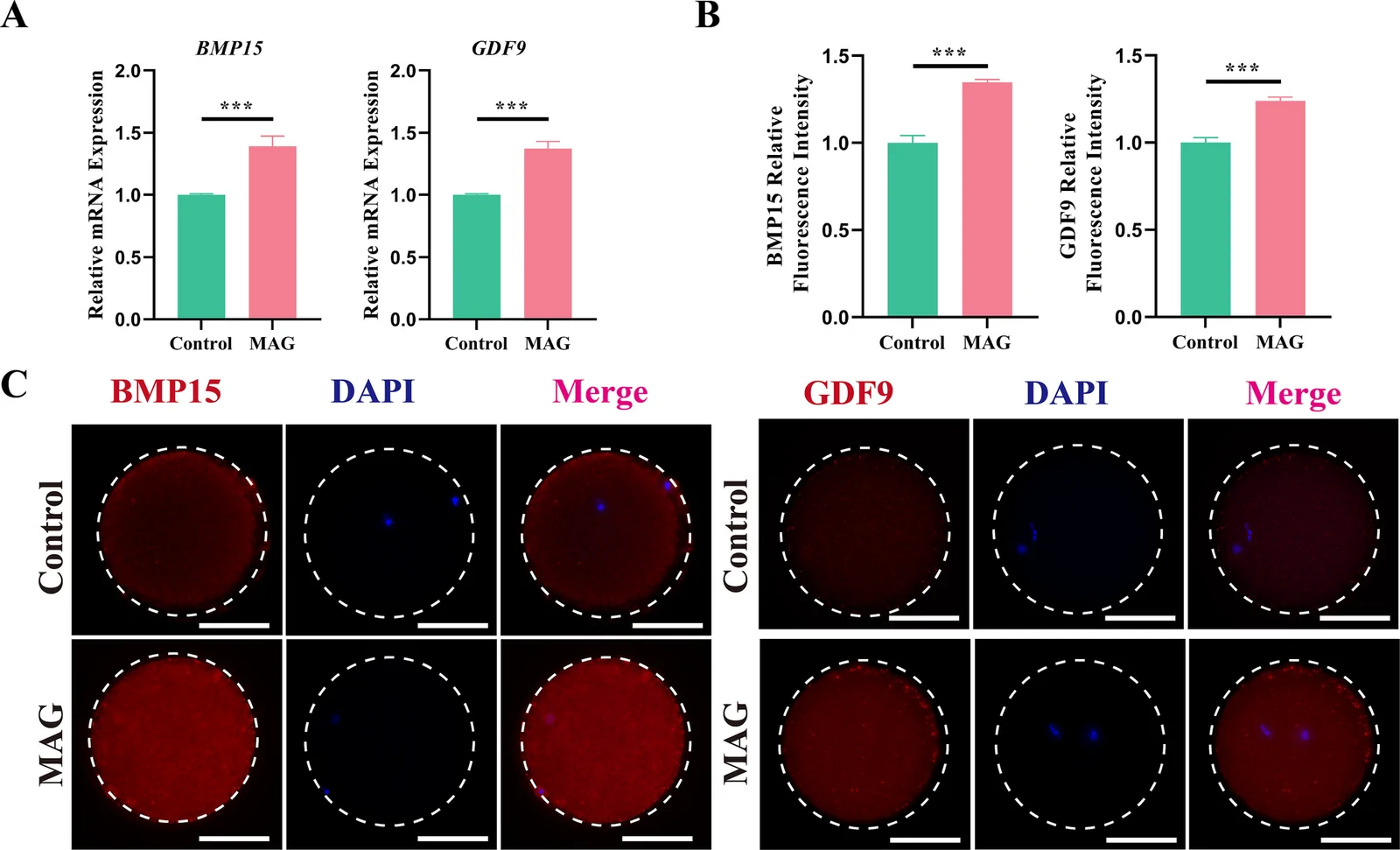
Effect of MAG on maturation of yak oocytes. **A** Relative mRNA expression levels of *BMP15* and *GDF9*. **B** Relative fluorescence expression level of BMP15 and GDF9 protein in yak oocytes. **C** Immunofluorescence staining analysis of BMP15 and GDF9 protein in yak oocytes. Scale bar = 50 μm. Data are presented as mean ± SEM from three independent biological replicates (*n* = 3). ^*^P < 0.05, ^**^P < 0.01, ^***^P < 0.001. BMP15, Bone morphogenetic protein 15; GDF9, Growth differentiation factor 9

To evaluate the effects of MAG on yak oocytes, IVM medium was supplemented with different concentrations of MAG (0, 0.5, 1, 5, and 10 μmol/L). As shown in Table 2, treatment with 1 μmol/L MAG significantly increased the oocyte maturation rate by 6.21% compared with the control group (*P* < 0.05). Therefore, 1 μmol/L MAG was selected for subsequent experiments. The expression of the oocyte maturation-related genes *GDF9* and *BMP15* was further examined, with qPCR analysis showing that their mRNA expression levels were significantly increased by the MAG treatment (*P* < 0.001, Fig. 2A). Consistently, immunofluorescence analysis showed markedly enhanced protein expression of GDF9 and BMP15 in the MAG-treated group (*P* < 0.001, Fig. 2B and C).

**Table 2:** Effects of different doses of MAG supplementation on yak oocyte maturation

Group, μmol/L	Number of oocytes	Number of maturation oocytes	Maturation rate, %
0	600	420	70.00 ± 3.06^b^
0.5	600	441	74.35 ± 2.36^ab^
1	600	485	80.83 ± 1.64^a^
5	600	428	71.33 ± 1.76^ab^
10	600	407	67.83 ± 2.02^b^

Numbers of oocytes and maturation oocytes are presented as total counts pooled from three independent biological replicates. 200 oocytes were included per treatment group in each of three independent biological replicates (*n* = 3). Maturation rates are presented as mean ± SEM. Different letters indicate significant differences (*P* < 0.05)

### Effects of MAG on cumulus cell expansion and expression of expansion-related genes

**Fig. 3:**
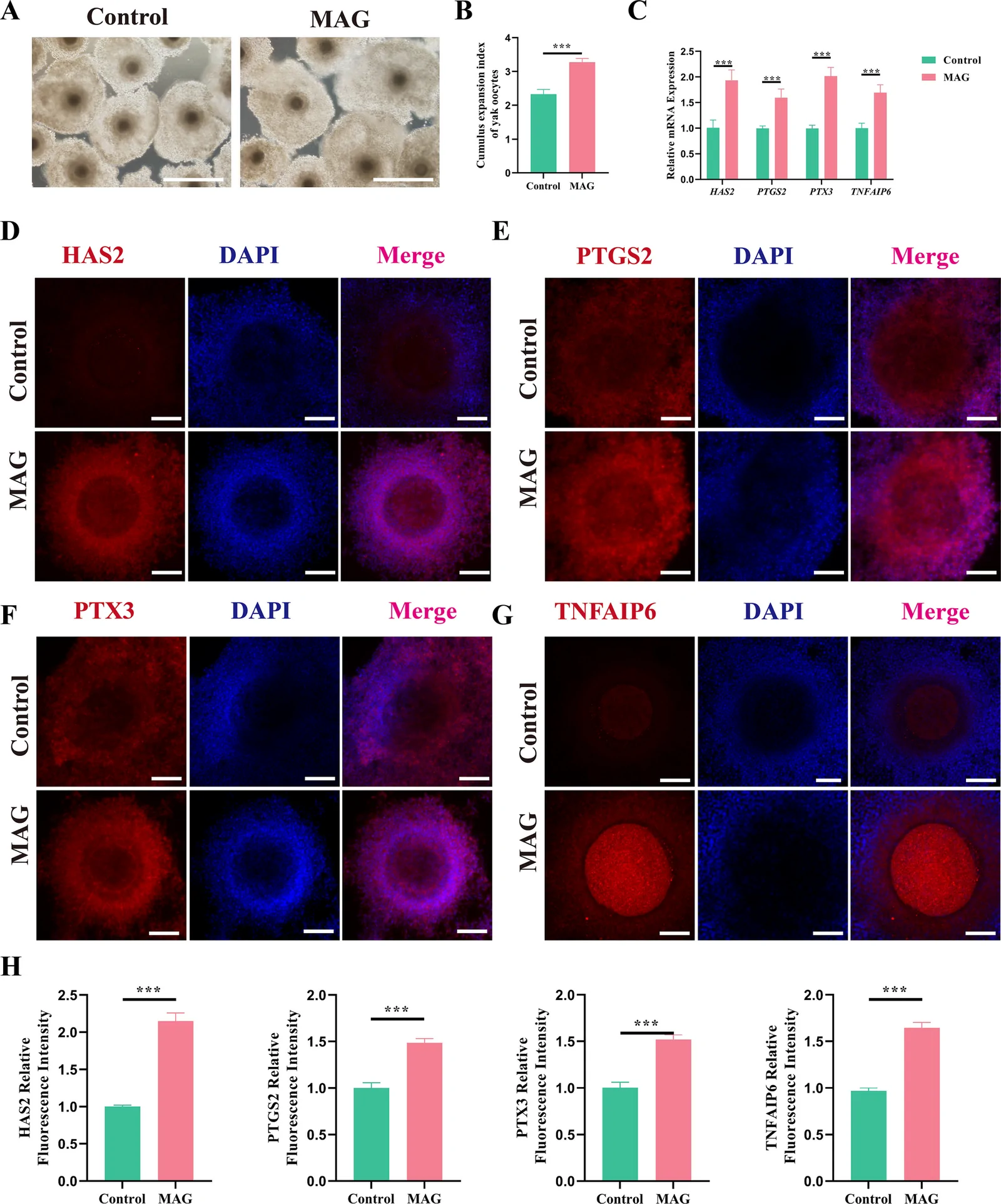
Effects of MAG on cumulus cell expansion and expression of expansion-related genes. **A** Morphological images of oocytes after 24 h, scale bar = 200 μm. **B** The effect of MAG on the CEI of yak oocytes. **C** Relative mRNA expression levels of cumulus expansion-related factors. **D–G** Immunofluorescence staining analysis of HAS2, PTGS2, PTX3 and TNFAIP6 protein in yak COCs. Scale bar = 50 μm. **H** Relative fluorescence expression level of HAS2, PTGS2, PTX3 and TNFAIP6 in yak COCs. Data are presented as mean ± SEM from three independent biological replicates (*n* = 3). ^*^P < 0.05, ^**^P < 0.01, ^***^P < 0.001. HAS2, Hyaluronan synthase 2; PTGS2, Prostaglandin-endoperoxide synthase 2; PTX3, Pentraxin 3; TNFAIP6, Tumor necrosis factor alpha-induced protein 6

Cumulus cell expansion is a critical event during oocyte maturation. As shown in Fig. 3A and B, the CEI was significantly higher in the MAG-treated group compared with the control group (*P* < 0.001). qPCR analysis demonstrated that the mRNA expression levels of key cumulus expansion–related genes, including *HAS2*, *PTGS2*, *PTX3*, and *TNFAIP6*, were significantly upregulated following MAG treatment (*P* < 0.001; Fig. 3C). Immunofluorescence analysis further confirmed increased expression of HAS2, PTGS2, PTX3, and TNFAIP6 proteins in the MAG group (*P* < 0.001, Fig. 3D–H).

### Effects of MAG on the antioxidant capacity of yak oocytes

**Fig. 4:**
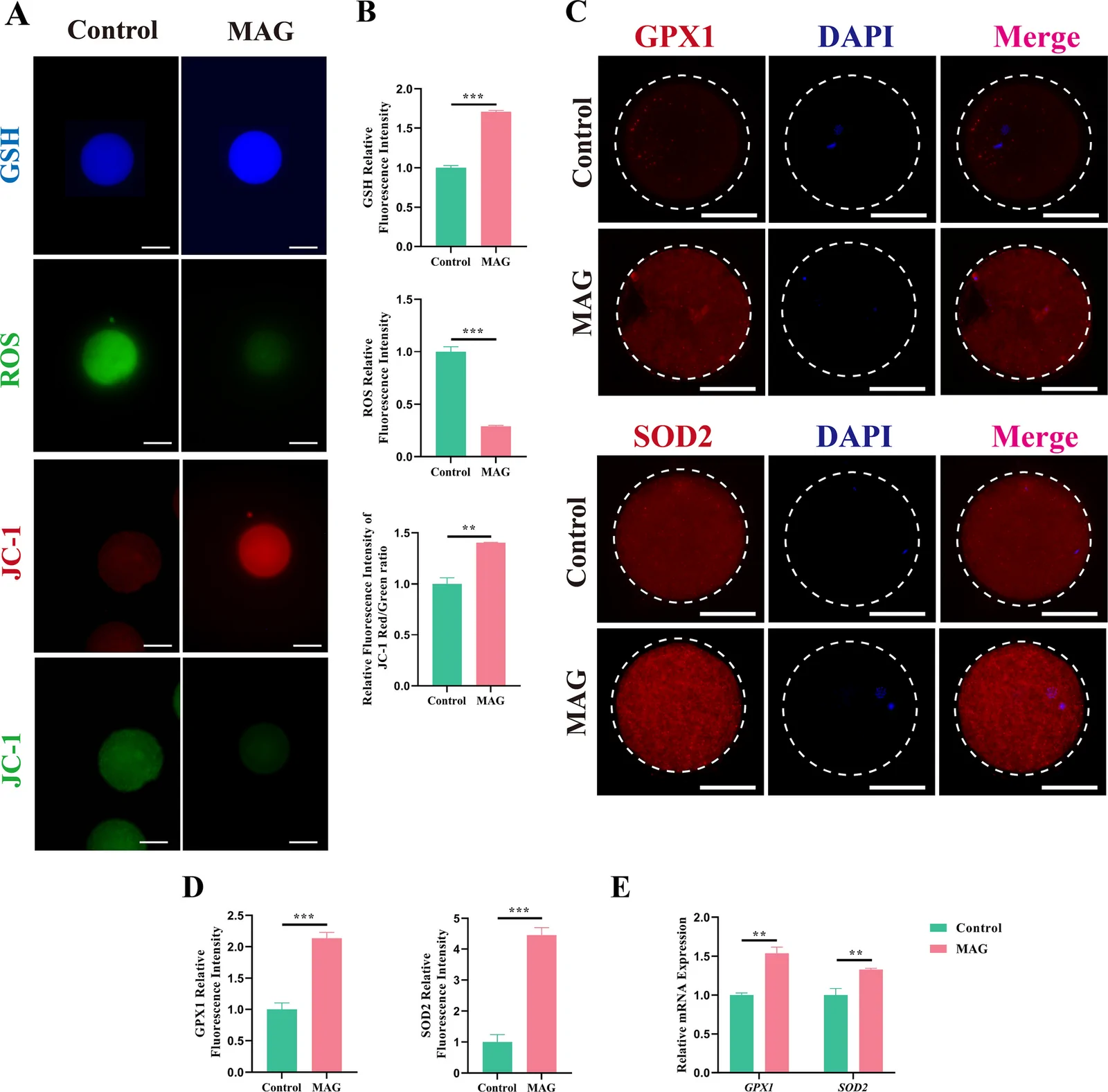
Effect of adding MAG on oxidative stress status in yak oocytes. **A** Representative images of MII oocytes stained with GSH, ROS and JC-1. Scale bar = 50 μm. **B** Statistical analysis of ROS, GSH and JC-1 Red/Green ratio relative fluorescence intensity. **C** Immunofluorescence staining analysis of GPX1 and SOD2 protein in yak oocytes. Scale bar = 50 μm. **D** Relative fluorescence expression level of GPX1 and SOD2 protein. **E** Relative mRNA expression levels of *GPX1* and *SOD2*. Data are presented as mean ± SEM from three independent biological replicates (*n* = 3). ^*^P < 0.05, ^**^P < 0.01, ^***^P < 0.001. GPX1, Glutathione peroxidase 1; SOD2, Superoxide dismutase 2

To explore whether MAG improves oocyte maturation by modulating oxidative status, intracellular ROS and GSH levels were assessed. As shown in Fig. 4A, MAG-treated yak oocytes exhibited significantly lower ROS fluorescence intensity compared with the control group (*P* < 0.001, Fig. 4A and B). In contrast, GSH fluorescence intensity was significantly increased in the MAG-treated group (*P* < 0.001; Fig. 4A and B). Similarly, we observed that the MAG-treated group exhibited a higher mitochondrial membrane potential compared with the control group (*P* < 0.01; Fig. 4A and B). Immunofluorescence analysis confirmed increased expression of GPX1 and SOD2 proteins in the MAG-treated oocytes (*P* < 0.001, Fig. 4C and D). Furthermore, the qPCR analysis showed that MAG significantly upregulated the mRNA expression of antioxidant-related genes *GPX1* and *SOD2* (*P* < 0.01; Fig. 4E).

### Effects of MAG on apoptosis in yak oocytes

**Fig. 5:**
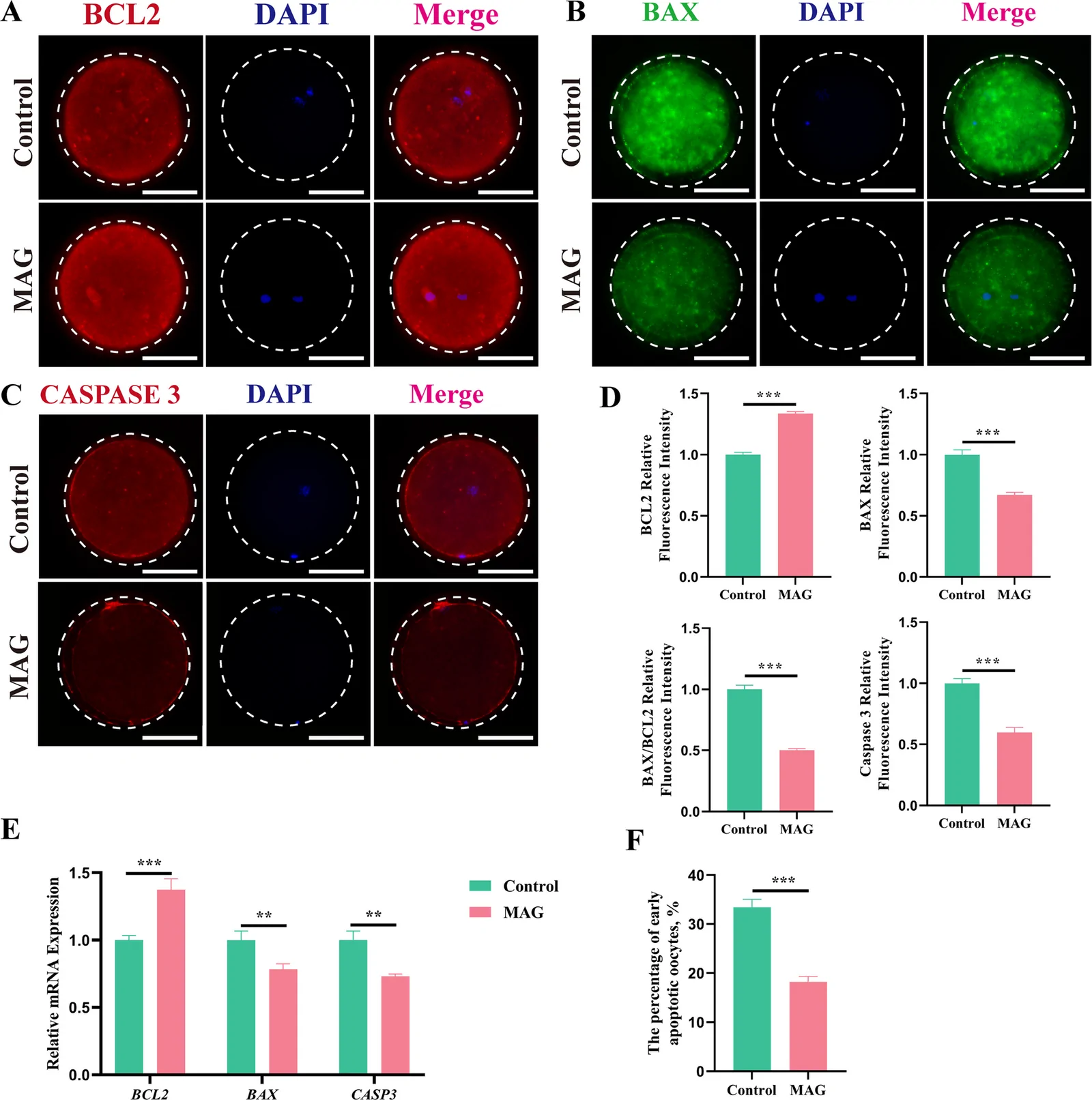
Effect of adding MAG on apoptosis in yak oocytes. **A**–**C** Immunofluorescence staining analysis of BCL2, BAX and CASP3 protein in yak oocytes. Scale bar = 50 μm. **D** Relative fluorescence intensity of BCL2, BAX, and Caspase 3 proteins, and the BAX/BCL2 ratio. **E** Relative mRNA expression levels of *BCL2*, *BAX*, and *CASP3*. **F** Statistical analysis of percentage of early apoptosis of oocytes. Data are presented as mean ± SEM from three independent biological replicates (*n* = 3). ^*^P < 0.05, ^**^P < 0.01, ^***^P < 0.001. BCL2, B-cell lymphoma 2; BAX, BCL2-associated X protein

To investigate the effects of MAG on apoptosis in yak oocytes, the gene and protein expression levels of BCL2, BAX, and CASP3 were evaluated. As shown in Fig. 5, compared with the control group, MAG supplementation significantly increased the mRNA and protein expression levels of BCL2 (*P* < 0.001, Fig. 5A, D, and E) while significantly decreasing the mRNA and protein expression levels of BAX (*P* < 0.01 and *P* < 0.001, Fig. 5B, D, and E), resulting in a reduced BAX/BCL2 ratio (*P* < 0.001, Fig. 5D). In addition, the expression of CASP3 gene and protein were markedly decreased in the MAG-treated group (*P* < 0.01 and *P* < 0.001, Fig. 5C–E). Furthermore, the early apoptosis rate of yak oocytes was evaluated. As shown in Fig. 5F, MAG supplementation significantly reduced the early apoptosis rate of yak oocytes by 45.50% compared with the control group (*P* < 0.001, Fig. 5F).

### Effects of MAG on lipid metabolism in yak oocytes

**Fig. 6:**
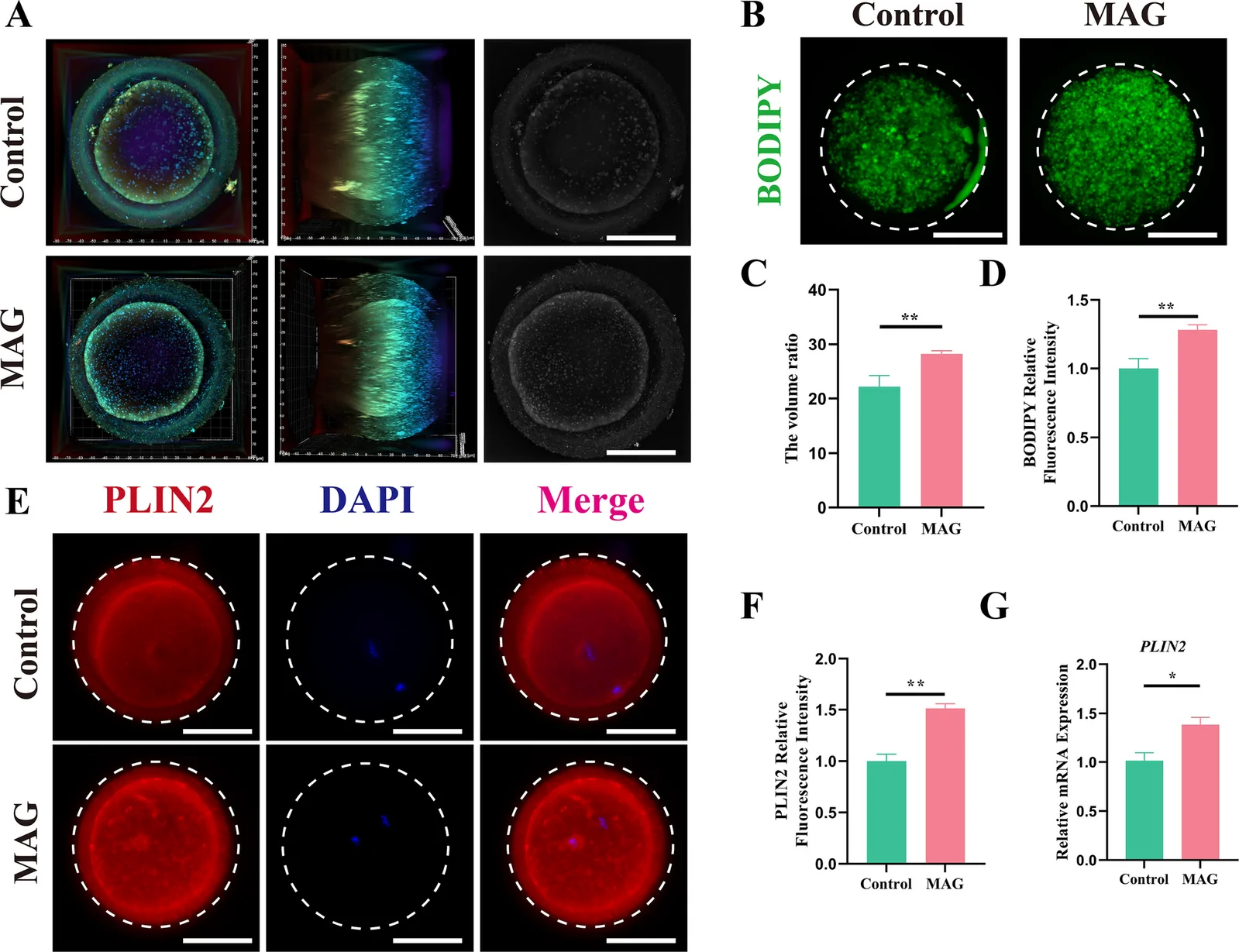
Effect of adding MAG on lipid metabolism in yak oocytes. **A** Three-dimensional holotomography image of the yak oocytes. Scale bar = 50 μm. **B** Representative images of LDs staining using BODIPY 493/503 in the yak oocytes. Scale bar = 50 μm. **C** Lipid droplet volume analyzed using 3D hologram microscopy. **D** Relative fluorescence expression level of BODIPY 493/503. **E** Immunofluorescence staining analysisi of PLIN2 protein. Scale bar = 50 μm. **F** Relative fluorescence expression level of PLIN2 protein in yak oocytes. **G** Relative mRNA expression levels of *PLIN2*. Data are presented as mean ± SEM from three independent biological replicates (*n* = 3). ^∗^*P* < 0.05, ^∗∗^*P* < 0.01. PLIN2, Perilipin 2

Lipid content is essential for oocyte maturation and early embryonic development. Lipid droplet analysis was performed through three-dimensional imaging of mature yak oocytes using holographic microscopy (Fig. 6A and Supplementary Materials 2–5), which showed that MAG supplementation significantly increased the lipid droplet volume ratio in yak oocytes by 27.09% compared with the control group (*P* < 0.01, Fig. 6C). BODIPY 493/503 staining revealed that MAG supplementation significantly increased the intracellular lipid content of yak oocytes compared with the control group (*P* < 0.01, Fig. 6B and D). The immunofluorescence analysis revealed that MAG supplementation significantly increased PLIN2 protein expression compared with the control group (*P* < 0.01, Fig. 6E and F). This result was further confirmed by qPCR analysis, which showed enhanced mRNA expression levels of *PLIN2* in MAG-treated oocytes (*P* < 0.05, Fig. 6G).

### Network pharmacology analysis identifies PPAR signaling as a key pathway of MAG

**Fig. 7:**
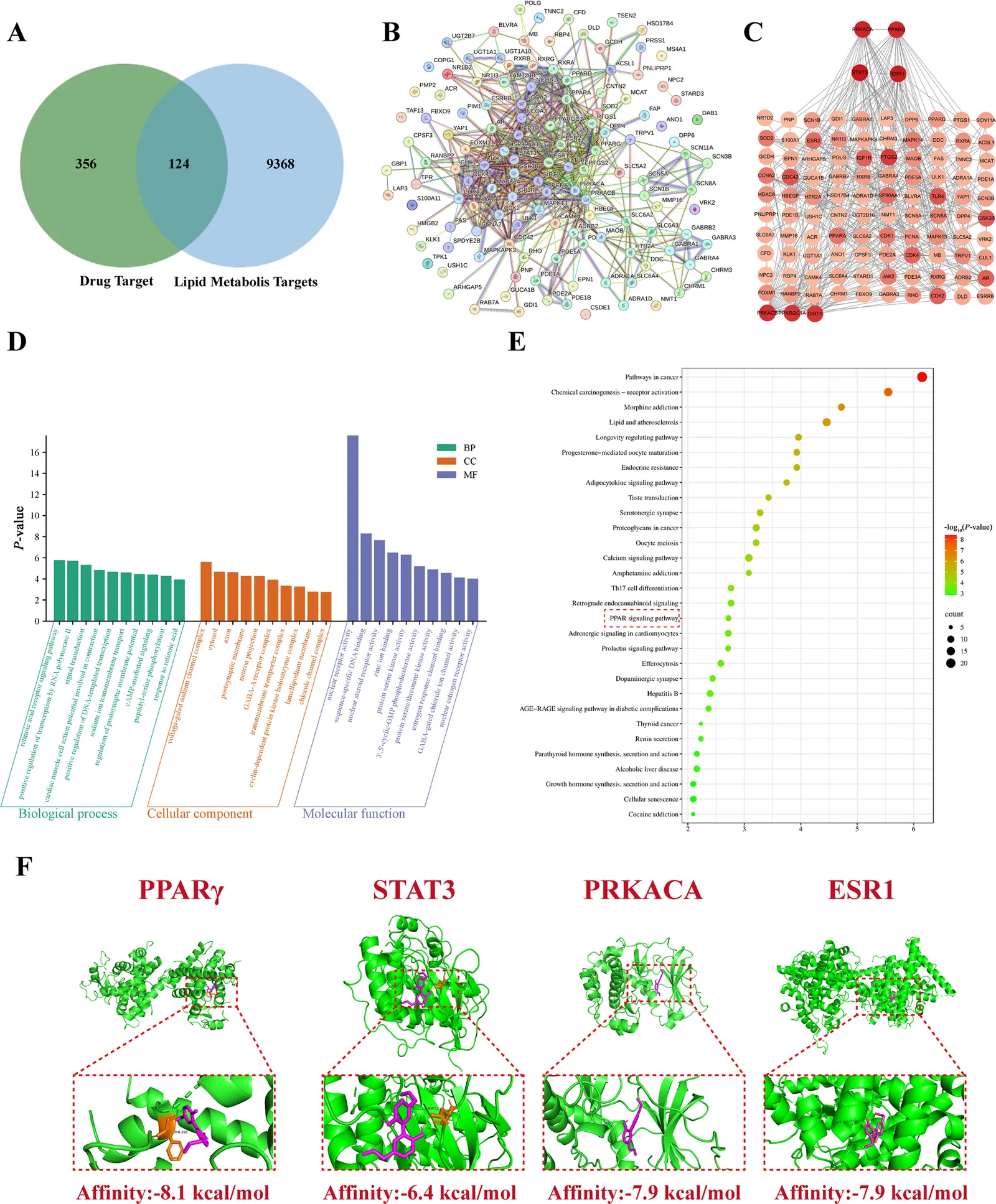
MAG network pharmacology analysis results. **A** Venn diagram of the active compounds of MAG and bovine lipid metabolism target. **B** PPI network of bovine lipid metabolism target and MAG. **C** PPI network diagram based on degree value. **D** GO function enrichment analysis. **E** KEGG pathway enrichment analysis. **F** Molecular docking diagram of MAG and targets. PPI, Protein–protein interaction; GO, Gene Ontology; KEGG, Kyoto Encyclopedia of Genes and Genomes; PPARγ, Peroxisome proliferator-activated receptor gamma; STAT3, Signal transducer and activator of transcription 3; PRKACA, Protein kinase cAMP-activated catalytic subunit alpha; ESR1, Estrogen receptor 1

Network pharmacology analysis was performed to explore the potential molecular mechanisms underlying MAG-regulated lipid metabolism. Through this, 356 drug targets and 9,368 lipid metabolic targets were obtained from the databases, with 124 overlapping targets identified (Fig. 7A). These 124 overlapping targets were entered into the STRING database to construct a PPI network, which was subsequently imported into Cytoscape 3.9.0 for visualization (Fig. 7B). Topological analysis identified PPARγ, STAT3, PRKACA, and ESR1 as the core targets (Fig. 7C). GO enrichment analysis revealed that MAG-associated targets were primarily enriched in retinoic acid signaling pathways, the voltage-gated sodium channel complex and nuclear receptor activity (Fig. 7D). KEGG pathway enrichment analysis indicated significant involvement of the PPAR, calcium, adipocytokine, and prolactin signaling pathways (Fig. 7E). The molecular docking results showed that MAG can form hydrogen bonds with PPARγ and STAT3, with the binding affinity between MAG and PPARγ being the strongest (−8.1 kcal/mol, Fig. 7F).

### MAG improves oocyte quality through a PPARγ-dependent mechanism

**Fig. 8:**
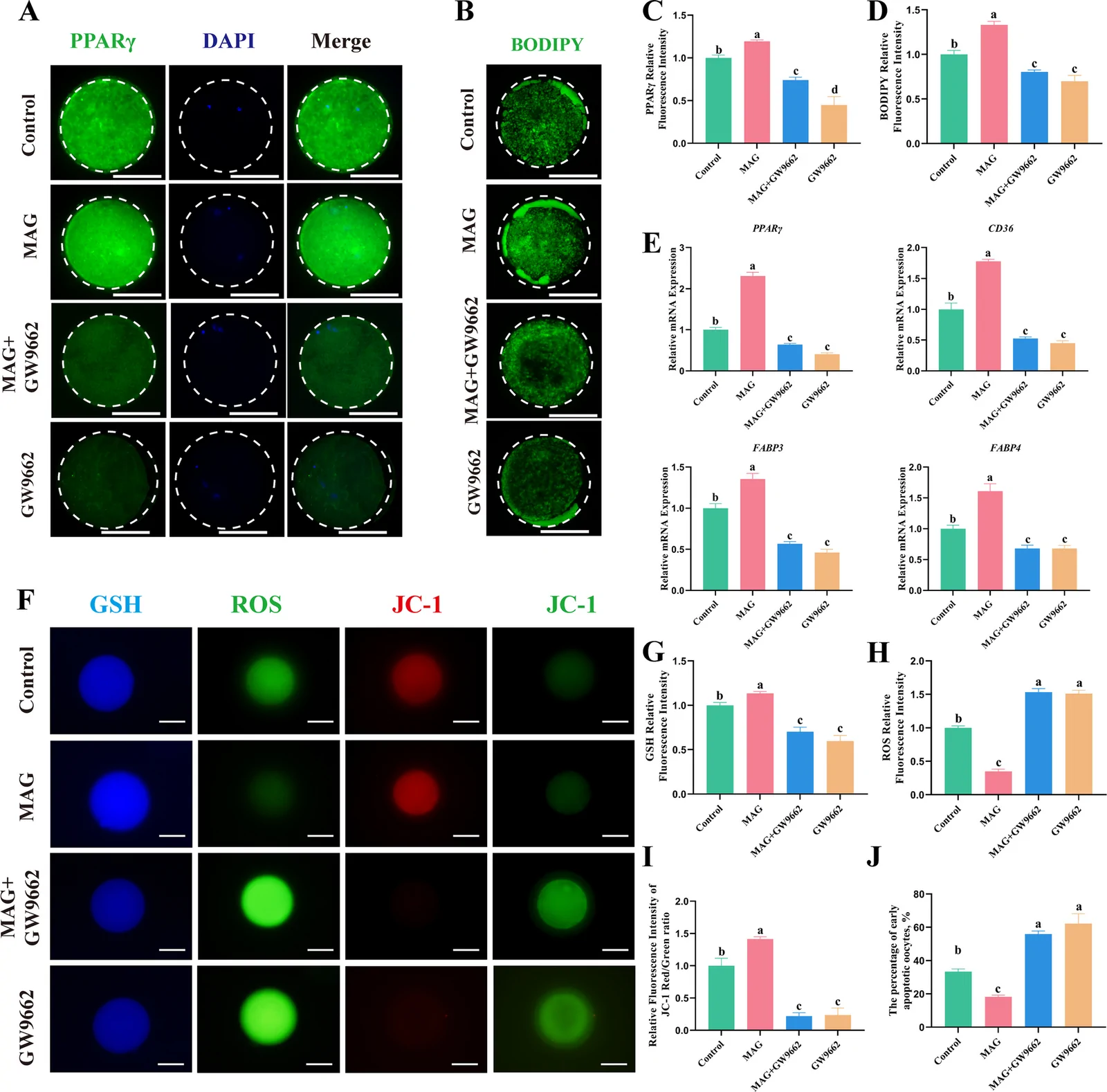
MAG improves oocyte quality through a PPARγ-dependent mechanism. **A** Immunofluorescence staining analysis of PPARγ protein in yak oocytes. Scale bar = 50 μm. **B** Representative images of LDs staining using BODIPY 493/503 in yak oocytes. **C** and **D** Relative fluorescence expression level of PPARγ and BODIPY 493/503. **E** Relative mRNA expression levels of *PPARγ*, *CD36*, *FABP3* and *FABP4*. **F** Representative images of MII oocytes stained with GSH, ROS, and JC-1. Scale bar, 50 μm. **G**–**I** Statistical analysis of ROS, GSH and JC-1 Red/Green ratio relative fluorescence intensity. **J** Statistical analysis of percentage of early apoptosis of oocytes. Data are presented as mean ± SEM from three independent biological replicates (*n* = 3). Different superscripts within columns indicate significant differences (*P* < 0.05). PPARγ, Peroxisome proliferator-activated receptor gamma; CD36, Cluster of differentiation 36; FABP3, Fatty acid-binding protein 3; FABP4, Fatty acid-binding protein 4

To investigate the involvement of PPARγ signaling in the beneficial effects of MAG on lipid metabolism, the PPARγ antagonist GW9662 was added during IVM. As shown in Fig. 8A, MAG treatment significantly increased PPARγ protein expression compared with the control group (*P* < 0.05), whereas GW9662 treatment alone significantly reduced PPARγ expression (*P* < 0.05). Co-treatment with MAG and GW9662 markedly attenuated the MAG-induced increase in PPARγ protein levels (*P* < 0.05). Consistent with these findings, MAG significantly increased the intracellular lipid content and upregulated the mRNA expression of *PPARG*, *CD36*, *FABP3*, and *FABP4* compared with the control group (*P* < 0.05, Fig. 8B–E). In contrast, the GW9662 treatment alone reduced both lipid accumulation and the expression of these lipid metabolism–related genes. Furthermore, the stimulatory effects of MAG on lipid content and gene expression were significantly attenuated in the MAG + GW9662 group (*P* < 0.05, Fig. 8B–E). The GW9662 treatment adversely affected intracellular redox status. Compared with the control group, GW9662 significantly increased ROS levels, while decreasing the GSH content and mitochondrial membrane potential (*P* < 0.05). Notably, co-treatment with MAG and GW9662 largely abolished the beneficial effects of MAG on oxidative stress, apoptosis, and mitochondrial function, as evidenced by increased ROS levels and the percentage of early apoptotic oocytes, together with a reduced GSH content and mitochondrial membrane potential, compared to the MAG group (*P* < 0.05, Fig. 8F–J).

### MAG improves embryonic development and blastocyst quality through a PPARγ-dependent mechanism

**Fig. 9:**
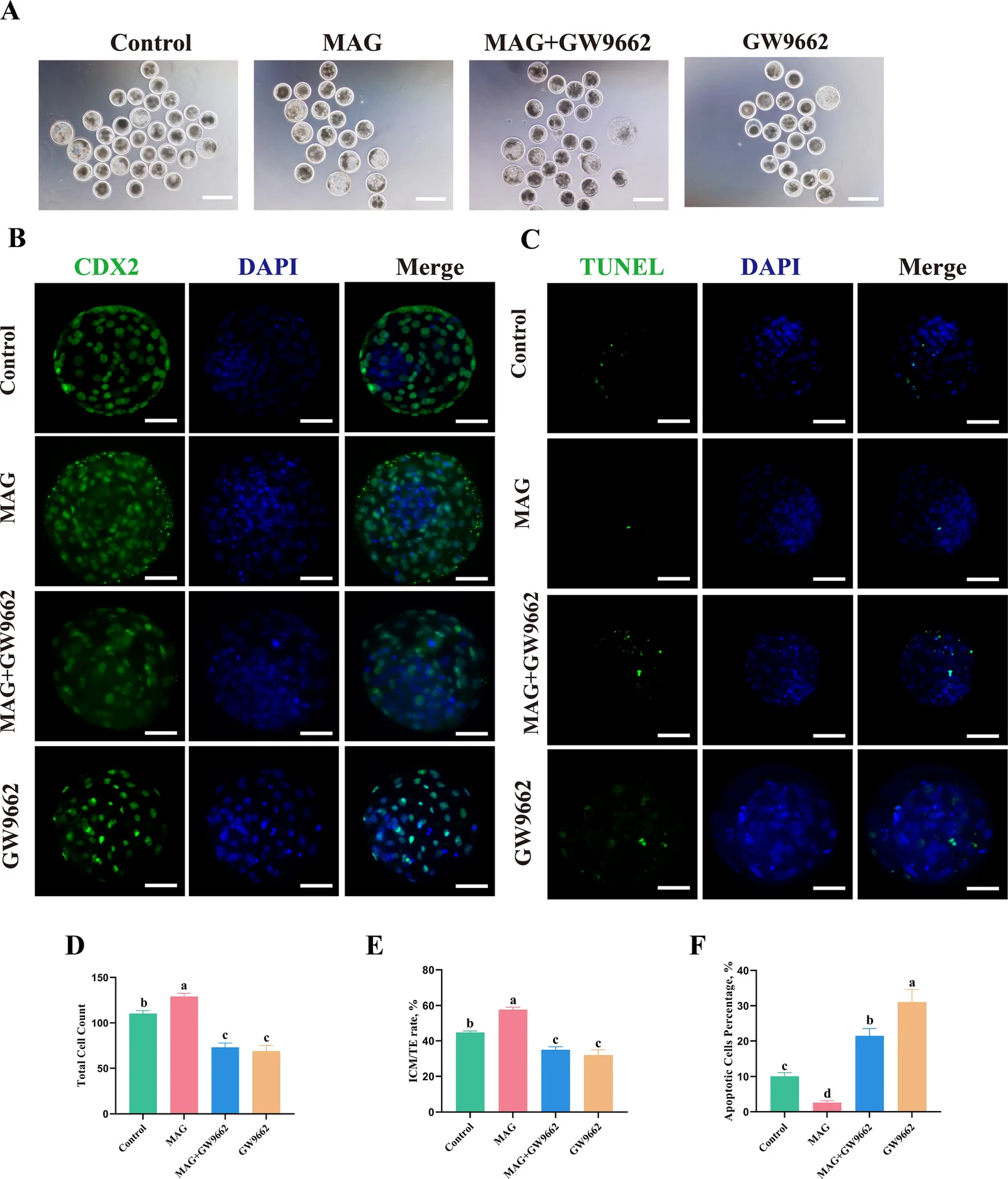
MAG improves embryonic development and blastocyst quality through a PPARγ-dependent mechanism. **A** Representative images of early embryos derived from in vitro fertilized oocytes. Scale bar = 200 μm. **B** Immunofluorescence staining analysis of CDX2 protein in yak oocytes. Scale bar = 50 μm. **C** TUNEL staining assessing apoptosis in yak blastocyst. **D** Statistical analysis of total cell number. Scale bar = 50 μm. **E** ICM/TE ratio in yak blastocyst. **F** Statistical analysis of the percentage of apoptotic cells. Data are presented as mean ± SEM from three independent biological replicates (*n* = 3). Different superscripts within columns indicate significant differences (*P* < 0.05). CDX2, Caudal-type homeobox 2; ICM, Inner cell mass; TE, Trophectoderm

To evaluate whether PPARγ signaling is involved in the beneficial effects of MAG on embryonic development, IVF and embryo culture were performed following oocyte maturation. As shown in Fig. 9A and Table 3, MAG supplementation significantly increased the cleavage rate and blastocyst formation rate compared with the control group (*P* < 0.05). In contrast, treatment with GW9662 alone significantly reduced blastocyst formation compared with the control group (*P* < 0.05). Furthermore, co-treatment with MAG and GW9662 significantly attenuated the promotive effects of MAG on embryonic development, resulting in lower cleavage and blastocyst formation rates than those observed in the MAG group (*P* < 0.05). The immunofluorescence analysis demonstrated that blastocysts derived from the MAG-treated group exhibited a significantly higher total cell number and ICM/TE ratio, together with a lower apoptotic index, compared to the control group (*P* < 0.05, Fig. 9B–F). Conversely, the GW9662 treatment alone impaired blastocyst quality, as evidenced by a reduced total cell number and ICM/TE ratio, accompanied by an increased apoptotic index (*P* < 0.05, Fig. 9B–F). Importantly, co-treatment with MAG and GW9662 largely reversed the beneficial effects of MAG on blastocyst quality, resulting in a decreased total cell number and ICM/TE ratio and increased apoptotic index compared to the MAG group (*P* < 0.05, Fig. 9B–F).

**Table 3:** Effect of GW9662 on subsequent development of oocytes following IVF

Group	Number of zygotes	Number of cleaved embryos	Number of blastocysts	Cleaved embryo rate, %	Blastocyst rate, %
Control	300	177	53	59.00 ± 1.73^b^	17.67 ± 1.20^b^
MAG	300	211	75	70.33 ± 0.88^a^	25.00 ± 1.15^a^
MAG + GW662	300	122	29	40.67 ± 1.45^c^	10.00 ± 0.58^c^
GW662	300	120	27	40.00 ± 2.65^c^	9.00 ± 1.15^c^

Numbers of zygotes, cleaved embryos, and blastocysts are presented as total counts pooled from three independent biological replicates. 100 zygotes were cultured per treatment group in each biological replicate. Cleavage and blastocyst formation rates are presented as mean ± SEM from three independent biological replicates (*n* = 3). Different letters indicate significant differences (*P* < 0.05)

## Discussion

The yak is an economically important livestock species on the Qinghai–Tibet Plateau; however, its relatively low reproductive efficiency remains a major constraint to sustainable production. In vitro embryo production has been widely applied to improve reproductive performance in domestic animals, and the acquisition of high-quality oocytes during IVM is a critical prerequisite for successful fertilization and subsequent embryonic development. In the present study, we demonstrate that supplementation with MAG during IVM significantly enhances yak oocyte developmental competence and blastocyst quality, primarily through the regulation of oxidative homeostasis and lipid metabolism via PPARγ signaling.

Oocyte maturation is highly dependent on bidirectional communication between the oocyte and surrounding CCs. In this study, MAG supplementation significantly enhanced the CEI and upregulated key cumulus expansion–related genes, including *HAS2*, *PTGS2*, *PTX3*, and *TNFAIP6*. Hyaluronan synthesis mediated by HAS2 is essential for extracellular matrix formation and structural stabilization of the cumulus–oocyte complex [36], while PTGS2-derived prostaglandin E2 plays a pivotal role in cumulus expansion and meiotic progression [37]. TNFAIP6 and PTX3 further stabilize the hyaluronan-rich matrix, and disruption of these components has been associated with impaired fertility [38]. Importantly, MAG treatment also significantly increased the mRNA expression levels of *GDF9* and *BMP15*, two oocyte-derived growth factors essential for follicular development and oocyte competence [39, 40]. These factors act synergistically to enhance cumulus cell function and promote cytoplasmic maturation. Collectively, our findings indicate that MAG strengthens oocyte–cumulus cell cooperation, thereby facilitating both nuclear and cytoplasmic maturation of yak oocytes.

Cytoplasmic maturation is closely associated with intracellular redox balance [41]. GSH is a key intracellular antioxidant and an established indicator of oocyte quality [42]; at low levels, the developmental ability of mature oocytes in vitro will be inhibited [43]. In the present study, MAG significantly increased GSH levels while reducing intracellular ROS accumulation. These changes were accompanied by the upregulation of antioxidant-related genes, including *GPX1* and *SOD2*, which are known to play critical roles in mitigating oxidative stress. ROS, like GSH, play crucial roles in cellular signal transduction and maintenance of redox homeostasis in vivo. By maintaining appropriate ROS levels and strengthening antioxidant defense mechanisms, oxidative stress-induced cellular damage and disease progression can be effectively prevented [44]. Mitochondrial dysfunction is a major source of excessive ROS generation, and elevated ROS levels can further impair mitochondrial activity, forming a vicious cycle that compromises oocyte quality [45]. Our results showed that MAG supplementation significantly enhanced mitochondrial membrane potential (ΔΨm), indicating improved mitochondrial function. Since ΔΨm is the driving force for ATP synthesis, its enhancement suggests that MAG may support the energetic demands of oocyte maturation. The observed improvement in mitochondrial function is likely linked to enhanced antioxidant capacity and reduced oxidative damage, ultimately contributing to improved oocyte developmental competence.

Lipid droplets are important energy reservoirs that provide fatty acids for mitochondrial β-oxidation during oocyte maturation and early embryonic development. However, increased lipid accumulation is not universally beneficial, as excessive lipid storage may induce lipotoxicity, promote lipid peroxidation, and impair mitochondrial function. Therefore, lipid homeostasis maintenance, rather than simple lipid accumulation, is essential for oocyte developmental competence. In the present study, MAG supplementation increased lipid droplet content while simultaneously enhancing PPARγ and PLIN2 expression, reducing oxidative stress, and improving mitochondrial function and embryonic development. These findings suggest that MAG may facilitate the balanced regulation of lipid storage and utilization, thereby support cellular energy metabolism while limiting oxidative damage during oocyte maturation.

Programmed cell death is an intrinsic regulatory mechanism during oocyte maturation and early embryonic development. However, excessive apoptosis negatively affects oocyte survival and developmental potential [46, 47]. In this study, MAG treatment significantly downregulated the expression of pro-apoptotic genes *BAX* and *CASP3*, suggesting that MAG protects yak oocytes from apoptosis during IVM. These findings are consistent with the improved redox balance and mitochondrial integrity observed in MAG-treated oocytes, as oxidative stress and mitochondrial dysfunction are well-established triggers of apoptosis.

LDs serve as important energy reservoirs that supply fatty acids for mitochondrial β-oxidation during oocyte maturation and early embryonic development [48]; however, increased lipid accumulation is not universally beneficial. Excessive lipid storage may induce lipotoxicity, promote lipid peroxidation, disrupt mitochondrial function, and ultimately impair oocyte quality [49]. Therefore, the maintenance of lipid homeostasis, rather than simple lipid accumulation, is critical for preserving oocyte developmental competence. In the present study, MAG supplementation increased LD content and the expression of lipid metabolism–related genes, including *PPARG*, *CD36*, *FABP3*, *FABP4*, and *PLIN2*. Notably, this increase in lipid storage was accompanied by reduced oxidative stress, enhanced mitochondrial function, and improved embryonic development. These findings suggest that MAG does not merely promote lipid accumulation but may facilitate a coordinated balance between lipid storage and utilization, thereby ensuring an adequate energy supply while preventing excessive oxidative damage during oocyte maturation. PPARs are ligand-activated nuclear receptors that play central roles in regulating lipid metabolism and energy homeostasis [50]. Among them, PPARγ is a key regulator of lipid uptake, storage, and metabolic adaptation [51]. In the present study, network pharmacology analysis and molecular docking suggested that PPARγ may be a potential target involved in the effects of MAG. The docking analysis predicted a stable interaction between MAG and PPARγ, with a binding energy of −8.1 kcal/mol. Although these in silico findings require further experimental validation, they provide preliminary mechanistic support for the involvement of PPARγ signaling. Functionally, MAG supplementation significantly increased PPARγ expression and promoted LD accumulation in yak oocytes. More importantly, pharmacological inhibition of PPARγ using GW9662 attenuated MAG-induced upregulation of PPARG and downstream lipid metabolism–related genes, reduced LD accumulation, and abolished the beneficial effects of MAG on redox homeostasis, mitochondrial function, oocyte quality, and embryonic development. These findings support the notion that PPARγ signaling is involved in the regulation of lipid metabolism and cellular homeostasis during MAG treatment. Previous studies have also demonstrated that MAG exerts antioxidant and cytoprotective effects through modulating PPARγ signaling in other biological systems [52]. Collectively, our findings suggest that the beneficial effects of MAG are associated with PPARγ-dependent regulation of lipid homeostasis rather than simply increased lipid storage. By promoting a balanced coordination between lipid accumulation, lipid utilization, mitochondrial function, and redox status, MAG may enhance the developmental competence of yak oocytes and support subsequent embryonic development.

During the preimplantation stage, the expression of pluripotency-related genes is closely associated with the embryo’s subsequent developmental potential [53]. CDX2 expression in the TE is one of the earliest known events in lineage specification [54]. After *Cdx2* gene knockout, *Cdx2*⁻^/^⁻ embryos are able to form a blastocoel; however, it cannot be maintained and is accompanied by morphological alterations in TE cells, whereas *Cdx2*⁺^/^⁺ and *Cdx2*⁺^/^⁻ embryos can form expanded blastocysts and hatch from the zona pellucida [55]. Consistent with these observations, MAG supplementation significantly increased CDX2 protein expression, accompanied by an increased total cell number and ICM/TE ratio, indicating that MAG improves blastocyst quality and supports normal TE development. The reduced apoptotic index observed in MAG-treated blastocysts further supports the protective role of MAG during early embryogenesis. Because successful preimplantation development requires coordinated regulation of lipid metabolism, mitochondrial function, and redox homeostasis, the improved embryo quality observed following MAG supplementation is likely attributable to enhanced oocyte cytoplasmic maturation established during IVM. Together with our findings that MAG promotes lipid metabolism, alleviates oxidative stress, and improves mitochondrial function through PPARγ signaling during oocyte maturation, these results suggest that involvement of the PPARγ pathway contributes to improved developmental competence of yak embryos by maintaining cellular metabolic homeostasis and reducing apoptosis.

## Conclusion

In conclusion, our findings demonstrate that MAG enhances yak oocyte maturation and subsequent embryonic development by improving lipid metabolism, alleviating oxidative stress, maintaining mitochondrial function, and reducing apoptosis during in vitro maturation. The attenuation of these beneficial effects by the PPARγ antagonist GW9662 supports the involvement of PPARγ signaling in the metabolic and cytoprotective effects of MAG. These findings provide new mechanistic insights into the regulation of oocyte developmental competence through PPARγ-dependent metabolic homeostasis and suggest that MAG is a promising supplement for improving yak in vitro embryo production.

## Supplementary Information

Supplementary Material 1: Fig. S1. Mature yak oocytes were subjected to Immunofluorescence staining with the IgG isotype control.

Supplementary Material 2: Video 1. Three-dimensional imaging video of mature yak oocytes in the control group.

Supplementary Material 3: Video 2. Holotomography video of mature yak oocytes in the control group.

Supplementary Material 4: Video 3. Three-dimensional imaging video of mature yak oocytes in the MAG group.

Supplementary Material 5: Video 4. Holotomography video of mature yak oocytes in the MAG group.

## Data Availability

No datasets were generated or analysed during the current study.

## Abbreviations

ACTB: Beta-actin
ARTs: Assisted reproductive technologies
BAX: BCL2-associated X protein
BCL2: B-cell lymphoma 2
BMP15: Bone morphogenetic protein 15
CD36: Cluster of differentiation 36
CDX2: Caudal-type homeobox 2
CEI: Cumulus expansion index
COCs: Cumulus–oocyte complexes
DAPI: 4′,6-Diamidino-2-phenylindole
ESR1: Estrogen receptor 1
FABP3: Fatty acid-binding protein 3
FABP4: Fatty acid-binding protein 4
GDF9: Growth differentiation factor 9
GO: Gene Ontology
GPX1: Glutathione peroxidase 1
GSH: Glutathione
HAS2: Hyaluronan synthase 2
ICM: Inner cell mass
IVF: In vitro fertilization
IVM: In vitro maturation
KEGG: Kyoto Encyclopedia of Genes and Genomes
LDs: Lipid droplets
MAG: Magnolol
MDA: Malondialdehyde
MII: Metaphase II
OMM: Oocyte maturation medium
PLIN2: Perilipin 2
PPI: Protein–protein interaction
PPARγ: Peroxisome proliferator-activated receptor gamma
PRKACA: Protein kinase cAMP-activated catalytic subunit alpha
PTGS2: Prostaglandin-endoperoxide synthase 2
PTX3: Pentraxin 3
ROI: Region of interest
ROS: Reactive oxygen species
SOD: Superoxide dismutase
SOD2: Superoxide dismutase 2
STAT3: Signal transducer and activator of transcription 3
TBP: TATA-box binding protein
TCMSP: Traditional Chinese Medicine Systems Pharmacology
TE: Trophectoderm
TGs: Triglycerides
TNFAIP6: Tumor necrosis factor alpha-induced protein 6
